# Supplementary material for: Factors Influencing Implementation, Sustainability and Scalability of Healthy Food Retail Interventions: A Systematic Review of Reviews
Source: Nutrients. 2022 Jan 11;14(2):294. doi: 10.3390/nu14020294 (PMC8780221; doi:10.3390/nu14020294)
Supplement: Supplementary file 1 [file nutrients-14-00294-s001.zip › nutrients-1541816-supplementary.pdf]

Supplementary data

<u>Table S1: Search terms used in the systematic search of the electronic databases.</u>

Search Strategy

Limits	Food retail outlet (type/ setting) *[Hedge1]*	Intervention foci *[Hedge 2]*	Outcomes [*Hedge 3]*
English Reviews only	"food industry" OR bodega* OR "corner store" OR "convenience store" OR "grocery store" OR diner* OR grocer* OR vending machine* OR "automatic food dispenser*" OR "fast food*" OR "take away" OR "dining room*" OR cafeteria* OR catering OR cafe* OR "ready to eat" OR food* OR beverage* OR drink* OR dispenser* OR supermarket* OR restaurant* OR shop* OR retail* OR store* OR "food environment" OR "food outlet*" OR "home delivery" OR ((Hospital OR college* OR school* OR university* OR education* OR office* OR store OR "sport centre"* OR "petrol station") NEAR/3 (food* OR drink* OR beverage* OR snack*))	product OR place* OR profile OR portion* OR pric* OR promotion OR priming OR prompt* OR proximity OR availability OR discount* OR voucher* OR incentive* OR bonus* OR reward* OR coupon* OR token* OR rebate* OR refund* OR access* OR display OR remov* OR layout OR strateg* OR advert* OR market* OR activit* OR initiative* OR program* OR "food quality" OR reformula* OR modif* OR adapt* OR recipe* OR product* OR "Point of purchase" OR "Point of sale"	implement* OR sustain* OR Scal* OR Engag* OR feasibil* OR "cost-benefit" OR "cost effective*" OR Fidelity OR Adoption OR retention OR accept* OR intervention* OR evaluat* OR "food choice" OR purchas*

Table S2: Definitions of implementation, sustainability and scalability

Implementation: demonstrated by the retailer's ability to execute a plan/intervention

- Acceptability: Perception among stakeholders that the intervention is useful or satisfactory
- Adoption: Intention, decision, or initiation to apply an intervention
- Appropriateness: Perceived fit, relevance, or compatibility of the intervention
- Engagement: In-depth involvement of the retailer in the intervention, extent of use of the intervention

Sustainability: demonstrated by the potential for maintenance of health benefits of an initiative over time

- Feasibility: Extent to which an intervention can be successfully implemented and economic feasibility
- Retention: Extent to which the benefits of the intervention can be retained
- Fidelity: Extent to which the intervention is delivered as intended

Scalability: demonstrated by the ability/potential to scale-up the intervention to other settings

- Cost-benefit: Extent to which the intervention is financially beneficial to the retailer in the long-run
- Cost effectiveness: Extent to which the intervention benefits and usage are a good value of money

Table S3: Quality Assessment

CRITERIA	1*. STUDY ELIGIBILITY CRITERIA						2**. IDENTIFICATION AND SELECTION OF STUDIES						3[#]. DATA COLLECTION AND STUDY APPRAISAL						4[##]. SYNTHESIS AND FINDINGS							5[@]. Describe whether conclusions were supported by the evidence			OVERALL RISK OF BIAS
**Author/year of publication**	1a	1b	1c	1d	1e	Overall score	2a	2b	2c	2d	2e	Overall score	3a	3b	3c	3d	3e	Overall score	4a	4b	4c	4d	4e	4f	Overall score	5a	5b	5c	
Adam and Jensen 2016[23]	PY ^	PY	PY	N	PY	Low Concern	Y	Y	Y	Y	PN	Low Concern	NI	Y	PY	Y	Y	High Concern	PY	PY	PY	Y	PY	PY	Low Concern	PY	Y	Y	Low Concern
Beltran and Romero, 2019[25]	PN	PY	PN	PY	PN	High Concern	Y	N	PY	PN	PY	High Concern	PY	PN	PN	N	NI	High Concern	PN	NI	PY	PY	N	N	High Concern	N	PN	PY	High Concern
Blake et al., 2019[16]	Y	Y	Y	PY	PY	Low Concern	Y	Y	Y	PN	Y	High Concern	Y	Y	PY	Y	Y	Low Concern	PY	Y	PY	Y	PY	PN	Low Concern	PY	PY	PY	Low Concern
Bucher et al., 2016[23]	Y	PY	Y	PY	PY	Low Concern	PY	PY	Y	PN	Y	Low Concern	PY	PY	Y	Y	Y	Low Concern	Y	Y	Y	Y	Y	PY	Low Concern	PY	PY	Y	Low Concern
Buttriss et al., 2004[26]	N	PN	PN	PN	PN	High Concern	N	PY	NI	PN	PN	High Concern	PN	PY	PY	PY	N	High Concern	NI	N	NI	NI	NI	PY	High Concern	N	N	PN	High Concern
Cameron et al., 2016[17]	N	Y	Y	Y	Y	Low Concern	Y	Y	PN	N	N	High Concern	NI	PY	PY	Y	PY	Low Concern	Y	NI	Y	NI	NI	N	High Concern	PN	Y	PN	High Concern
Escaron et al., 2013[5]	N	PY	PY	PN	PN	High Concern	N	PY	PY	PY	N	High Concern	N	PY	PY	N	N	High Concern	PY	N	PY	PY	PY	N	Low Concern	N	PY	PY	High Concern
Gittelsohn et al., 2012[18]	N	PY	PY	PY	PY	Low Concern	N	Y	N	PY	Y	Low Concern	Y	PY	PY	PN	PN	High Concern	PY	N	PY	PY	PY	N	Low Concern	PN	PY	PY	High Concern
Gittelsohn et al., 2013[27]	PN	PY	PN	PN	NI	High Concern	PN	PY	PN	NI	NI	High Concern	Y	PY	PY	NI	N	High Concern	PY	NI	PY	Y	PY	N	High Concern	N	PY	PY	High Concern
Gittelsohn et al., 2017[29]	PN	PY	PY	PY	PY	Low Concern	Y	Y	Y	Y	Y	Low Concern	Y	PY	PY	PY	PY	Low Concern	Y	NI	PY	PY	PY	PY	Low Concern	PY	PY	PY	Low Concern
Glanz et al., 2012[24]	PY	PY	PN	PY	PN	High Concern	Y	Y	PY	PY	NI	Unclear Concern	NI	PN	PN	N	N	High Concern	NI	PN	PY	PY	PN	PN	High Concern	N	PY	NI	High Concern

Grech and Allman-Farinelli 2015[30]	PY	PY	PY	PY	PN	Low Concern	Y	Y	PY	NI	NI	Unclear Concern	NI	PY	PY	Y	Y	Unclear Concern	PY	NI	Y	Y	PY	PY	Low Concern	PN	Y	Y	Unclear Concern
Henryks & Brimblecombe, 2016[24]	PY	PY	PN	Y	Y	Low Concern	PY	Y	PY	Y	NI	Unclear Concern	NI	N	NI	NI	N	High Concern	NI	NI	PY	Y	PY	NI	Unclear Concern	NI	Y	NI	Unclear Concern
Hillier-Brown et al., 2017[19]	PY	PN	Y	Y	PY	High Concern	Y	Y	Y	PY	PN	High Concern	PY	PN	PY	NI	N	High Concern	Y	NI	PY	Y	PY	N	High Concern	Y	PY	NI	High Concern
Hillier-Brown et al., 2017[28]	Y	PY	PY	PY	PY	Low Concern	PY	Y	PY	N	PY	High Concern	PY	PY	PY	Y	Y	Low Concern	Y	Y	PY	Y	PN	PY	Low Concern	N	PY	PY	Low Concern
Houghtaling et al., 2019[8]	PY	Y	Y	PY	PY	Low Concern	Y	PY	PY	PY	Y	Low Concern	Y	Y	PY	Y	Y	Low Concern	Y	Y	PY	Y	Y	PY	Low Concern	Y	Y	PY	Low Concern
Hua & Ickovics, 2016[31]	PY	PY	Y	PY	Y	Low Concern	PN	N	PY	PY	NI	High Concern	NI	Y	PY	NI	NI	High Concern	Y	NI	PY	Y	Y	N	High Concern	PN	Y	Y	High Concern
Kerins et al., 2020[9]	Y	Y	Y	PY	PY	Low Concern	PY	Y	Y	PY	PY	Low Concern	Y	Y	Y	Y	Y	Low Concern	Y	PY	Y	Y	PY	PY	Low Concern	Y	Y	Y	Low Concern
Kraak et al., 2017[35]	PY	PY	Y	Y	Y	Low Concern	Y	PY	PY	Y	NI	Low Concern	NI	Y	Y	NI	NI	High Concern	Y	NI	Y	Y	PY	N	Low Concern	PN	Y	Y	High Concern
Liberato et al., 2014[32]	PY	PY	Y	PY	PY	Low Concern	PY	N	PY	PY	Y	Low Concern	Y	Y	Y	Y	Y	Low Concern	Y	PY	PY	PY	PN	Y	Low Concern	PY	Y	Y	Low Concern
Mah et al., 2019[36]	PY	Y	Y	Y	Y	Low Concern	PY	PY	Y	Y	Y	Low Concern	PY	PY	PY	NI	NI	High Concern	Y	NI	PY	PY	PY	N	High Concern	PN	Y	Y	High Concern
Marcano-Olivier et al., 2020[20]	PN	PY	PY	PY	PY	Low Concern	Y	Y	PY	PY	Y	Low Concern	PY	PY	PY	Y	Y	Low Concern	Y	N	PY	Y	Y	PY	Low Concern	Y	PY	PY	Low Concern
Middel et al., 2019[10]	Y	Y	Y	PY	PN	Low Concern	Y	NI	Y	PY	Y	Low Concern	PN	Y	PY	PN	Y	High Concern	Y	Y	Y	Y	PY	PY	Low Concern	PY	Y	Y	Low Concern

von Philipsborn et al., 2019[21]	Y	Y	Y	PY	PY	Low Concern	Y	Y	Y	PY	Y	Low Concern	Y	Y	Y	Y	Y	Low Concern	Y	Y	Y	Y	Y	Y	Low Concern	Y	Y	Y	Low Concern
Wilson et al., 2016[22]	Y	Y	Y	Y	Y	Low Concern	Y	PY	PY	Y	Y	Low Concern	Y	Y	Y	Y	Y	Low Concern	Y	PY	PY	PY	PY	PY	Low Concern	Y	Y	Y	Low Concern

^PY: Probably Yes; Y: Yes; PN: Probably No; N: No; NI: No information; Yes= Low concern= High quality; No= High concern= Low quality; No information= Unclear concern= Moderate quality

1* STUDY ELIGIBILITY CRITERIA:	2**IDENTIFICATION AND SELECTION OF STUDIES	3#. DATA COLLECTION AND STUDY APPRAISAL	4##. SYNTHESIS AND FINDINGS	5@. Describe whether conclusions were supported by the evidence
1a. Did the review adhere to pre-defined objectives and eligibility criteria?	2a. Did the search include an appropriate range of databases/electronic sources for published and unpublished reports?	3a. Were efforts made to minimise error in data collection?	4a. Did the synthesis include all studies that it should?	5a. Did the interpretation of findings address all of the concerns identified in Domains 1 to 4?
1b. Were the eligibility criteria appropriate for the review question?	2b. Were methods additional to database searching used to identify relevant reports?	3b. Were sufficient study characteristics available for both review authors and readers to be able to interpret the results?	4b. Were all pre-defined analyses reported or departures explained?	5b. Was the relevance of identified studies to the review's research question appropriately considered?
1c. Were eligibility criteria unambiguous?	2c. Were the terms and structure of the search strategy likely to retrieve as many eligible studies as possible?	3c. Were all relevant study results collected for use in the synthesis?	4c. Was the synthesis appropriate given the nature and similarity in the research questions, study designs and outcomes across included studies?	5c. Did the reviewers avoid emphasizing results on the basis of their statistical significance?
1d. Were any restrictions in eligibility criteria based on study characteristics appropriate?	2d. Were restrictions based on date, publication format, or language appropriate?	3d. Were efforts made to minimise error in risk of bias assessment?	4d. Was between-study variation (heterogeneity) minimal or addressed in the synthesis?	
1e. Were any restrictions in eligibility criteria based on sources of information appropriate (e.g. publication	2e. Were efforts made to minimise error in selection of studies?	3e. Was risk of bias (or methodological quality) formally assessed using appropriate criteria?	4e. Were the findings robust, e.g. as demonstrated through funnel plot or sensitivity analyses?	

status or format, language, availability of data?				
			4f. Were biases in primary studies minimal or addressed in the synthesis?	
